# Effect of inverse kinematic alignment total knee arthroplasty on coronal alignment of the ankle joint in patients with varus knee deformity

**DOI:** 10.1007/s00402-024-05549-2

**Published:** 2024-09-13

**Authors:** Ittai shichman, Amer Hallak, Itay Ashkenazi, Yaniv Warschwaski, Aviram Gold, Nimrod Snir

**Affiliations:** 1 Division of Orthopedic Surgery, Tel-Aviv Sourasky Medical Center, Tel-Aviv, Israel; 2 Chief of Adult Reconstruction Unit, Division of Orthopedic Surgery, Tel Aviv Sourasky Medical Center, NYU Langone Orthopedic Center, 6 Weizman St. 6th Floor, Tel-Aviv, Israel

**Keywords:** Total knee arthroplasty, Patient specific alignment, Inverse kinematic alignment, Coronal alignment, Ankle alignment

## Abstract

**Introduction:**

Varus or valgus knee deformities influence ankle coronal alignments. The impact of Total Knee Arthroplasty (TKA) on ankle joint alignment has not been entirely illustrated. Inverse Kinematic Alignment (iKA) is a surgical philosophy that aims to restore soft tissue balance, function, and native anatomy within validated boundaries to restore restrictive native kinematics. Therefore, this study aimed to investigate the postoperative association of patient-specific alignment on the coronal alignment of the ankle in patients with varus knee deformity who underwent iKA TKA. We hypothesized that greater preoperative varus malalignments would correlate with significant postoperative ankle coronal alignment changes.

**Methods:**

This retrospective study of a prospective collected cohort assessed patients who underwent imageless navigation assisted robotic TKA using a single implant design for primary osteoarthritis between January 2022 and August 2023. Preoperative and postoperative full-length standing anteroposterior X-ray imaging was used to measure Hip-Knee-Ankle (HKA), Tibial Plafond Inclination (TPI), Talar inclination (TI), and Tibiotalar Tilt (TTT) angles. Patients were subsequently divided into groups of neutral varus) < 10°) and severe varus (≥ 10°) according to the preoperative HKA angle.

**Results:**

Significant changes in preoperative and postoperative HKA angles were found in the severe varus (14.5° vs. 6.4°, *p* < 0.001) group. Changes were also significant between preoperative and postoperative TPI and TI angles in the severe varus group; however, TTT did not reach statistical significance. Delta change from pre- to postoperative HKA was significantly higher for the severe varus group (8.1° vs. 0.8°, *p* < 0.019). Delta change of TPI, TI and TTT did not differ between groups.

**Conclusion:**

Coronal knee alignment after TKA affects coronal alignment of the ankle. iKA technique in TKA for varus knee deformity preserves or minimizes substantial coronal alignment changes of the ankle joint. These findings may add to the benefits reported for patient specific alignment TKA techniques.

**Level of evidence:**

III.

## Introduction

Total knee arthroplasty (TKA) is the acceptable surgical option for terminal-stage knee osteoarthritis over the past decades [1]. Although TKA is widely recognized as end-stage management for osteoarthritis, a noteworthy 11–25% of patients report dissatisfaction following TKA [1–3]. Of note, one of the most extensively studied methods for enhancing patient satisfaction post-TKA involves optimizing native knee alignment [4]. In particular, newer, modernized, approaches for restoring coronal alignment have been developed to improve clinical outcomes and satisfaction [2, 3]. Inverse Kinematic Alignment (iKA) is a variant of a patient specific coronal alignment technique which incorporates preoperative and intraoperative parameters that preserve the indigenous alignment and joint line inclination for each individual patient to reach a tailored outcome [4].

Though patient specific alignment (PSA) as risen in prominence, there is no clear-cut consensus on an optimal technique, as each surgery is planned according to surgeon experience preference and available institutional equipment [5]. In particular, it is important to consider the impacts of adjusting coronal knee alignment on hip and ankle alignment. Prior studies have established that 24–35% of patients receiving TKA may also have underlying ankle osteoarthritis [3, 6]. Furthermore, additional research has demonstrated that patients without simultaneous ankle osteoarthritis before their TKA may face a heightened likelihood of developing ankle osteoarthritis after the procedure, a finding that supports the notion that knee alignment significantly influences ankle alignment [3, 6].

Although several studies have been published to analyze the relationship between knee coronal alignment and the ankle, mainly following the mechanical approach, the exact effects of PSA and specifically iKA on the ankle have not been fully established [7]. Therefore, this study intended to measure the postoperative outcomes in the coronal alignment of the ankle joint relative to the knee in patients undergoing robotic TKA using iKA techniques. We hypothesized that greater preoperative varus malalignments would correlate with postoperative ankle coronal alignment change.

## Methods

### Patient population

Following approval from our institutional review board (0120-19-TLV), patients informed consent was waivered. Electronic medical records (EMR) were queried to identify patients who underwent iKA TKA using imageless robotic arm assisted total knee arthroplasties between January 2022 and August 2023. Patients were included if they were 18 years old or above, had a diagnosis of primary osteoarthritis (OA) of the knee and were undergoing elective, primary TKA. Patients were excluded if they had diagnoses of secondary OA (onset following inflammatory or infectious causes), post-traumatic arthritis, pathologic knee lesions, had a revision TKA or had any documented or evident past ankle trauma or surgical hardware. Additionally, simultaneous bilateral TKAs procedures and patients who underwent staged bilateral TKAs were excluded to avoid potential effect of TKA performed on one side on the contralateral side ankle coronal alignment. In addition, patients who did not complete or had low quality or malpositioned pre- and postoperative anterior-posterior (AP) standing weight-bearing full-body low-dose imaging were also excluded. We collected patient demographic data such as age, gender, laterality, body mass index (BMI), and American Society of Anesthesiologists (ASA) status. Of 200 patients who underwent the aforementioned surgery, a total of 123 patients met our inclusion criteria and were selected for the study. Patients were then categorized into two groups based on the degree of preoperative knee coronal alignments measured: Neutral (varus < 10°, *n* = 88) and severe varus (varus ≥ 10°, *n* = 36). Groups were then analyzed and subsequently compared.

A total of 123 patients met inclusion criteria. The cohort consisted of significantly more females (82/123, 66.7%, *p* = 0.002). The average age of the cohort was 69 years (standard deviation ± 9.6, range 40–90). Sixty-six (66/123, 53.7%) surgeries were performed on right knees and 57 (57/123, 46.3%) were performed on the left knees. The average BMI of the entire cohort was 30.6 (range: 19.3–49.6). There were no differences in demographic data between both groups [Table 1].

**Table 1:** Demographic distribution. ASA, American Society of Anesthesiologists; BMI, body mass index; SD, standard deviation

	Cohort (*N* = 123)	Study groups		*p*-value
		Neutral varus/valgus < 10° (*N* = 87)	Severe varus ≥ 10° (*N* = 36)	
Age (y)	69 (SD ± 9.6) Range (40–90)	68.6 Range (40–90)	72.5 Range (50–90)	0.146
Gender				
Male	41 (33.3%)	23 (26%)	18 (50%)	0.002
Female	82 (66.7%)	64 (74%)	18 (50%)	
Laterality				
Right	66 (53.7%)	52 (60%)	14 (39%)	0.779
Left	57 (46.3%)	35 (40%)	22 (61%)	
BMI (kg/m2)	30.6 (SD ± 5.7) Range (19.3–49.6)	30.9 Range (21.2–49.6)	29.9 Range (19.3–44.6)	0.289
ASA				
1	7	5	2	0.348
2	86	63	23	
3	27	18	9	
4	0	0	0	

### Radiographic assessment

All included patients have completed preoperative anterior-posterior (AP) standing weight-bearing full-body low-dose imaging and postoperatively 6 months. During the radiographic assessment, the foot rotation angle was managed using a reference foot template on the platform of the radiographic system. Additionally, appropriate rotational knee position (patellar facing forward) was confirmed before final acquisition of the full-limb AP radiographs. All radiographic images were digitally uploaded using a picture archiving and communication system (PACS). Patients with inadequate quality images or incomplete imaging were consequently excluded thereafter.

### Radiographic measurements

**Fig. 1:**
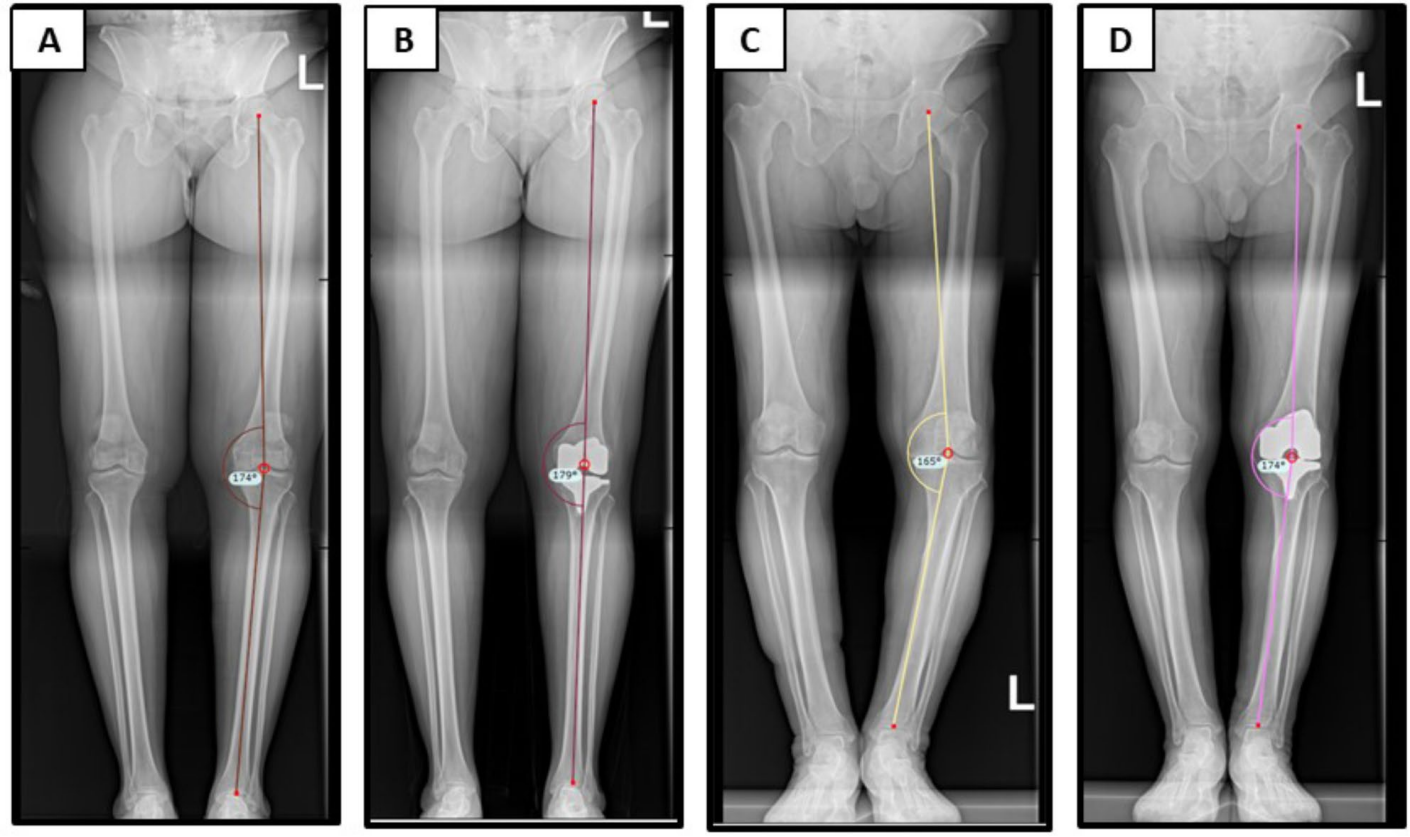
Full-body low-dose measurements. (**A**) neutral varus pre-operative hip-knee-ankle (**B**) neutral varus post-operative hip-knee-ankle (**C**) severe varus pre-operative hip-knee-ankle (**D**) severe varus post-operative hip-knee-ankle

**Fig. 2:**
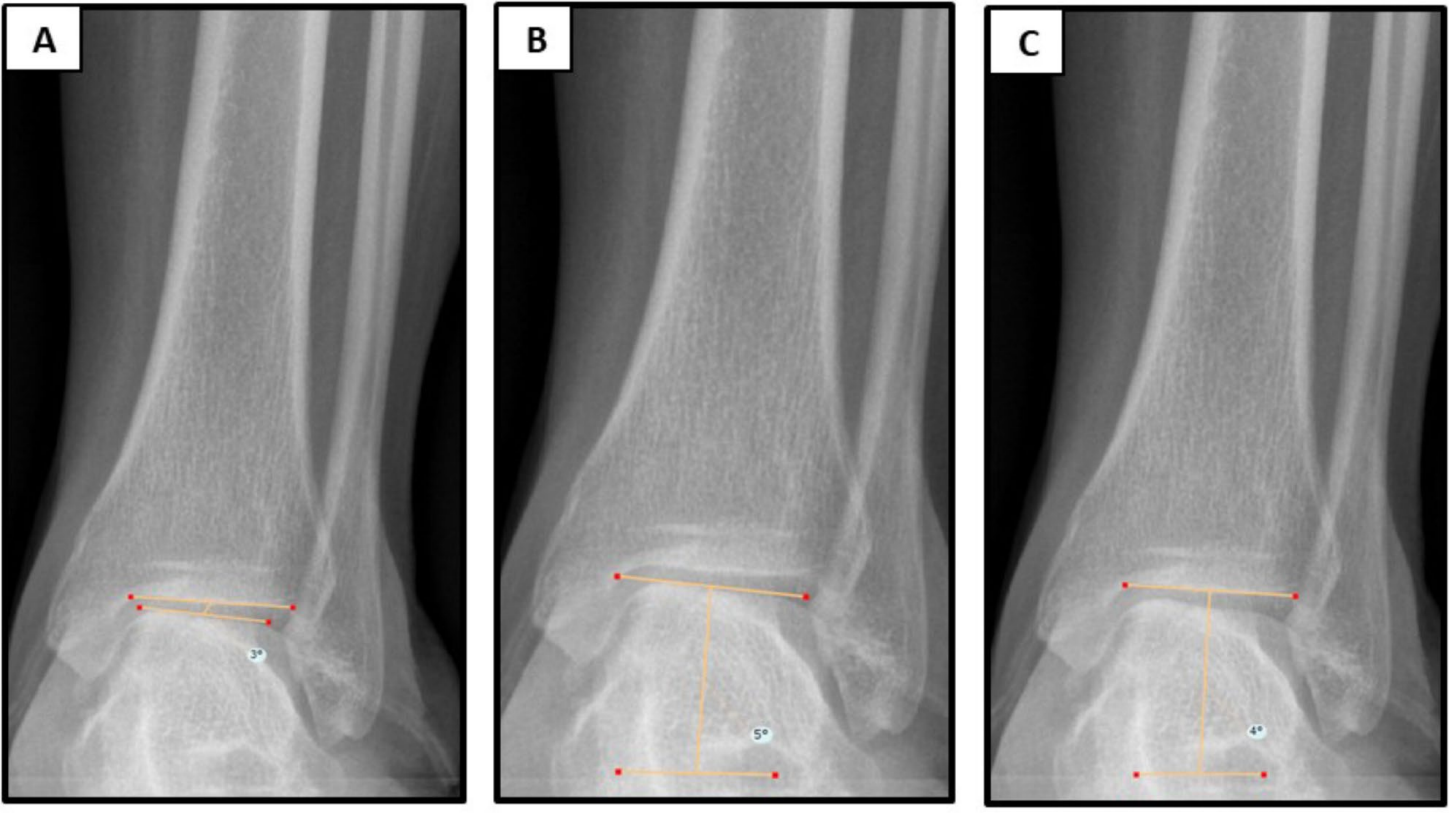
Magnification from pre-operative full-body low-dose measurements. (**A**) Tibiotalar tilt angle is the angle measured between the subchondral plate of the distal tibia articular surface and that of the talar dome. (**B**) Talar inclination is the angle measured between the talar dome and a vertical line to the ground. (**C**) Tibial plafond inclination is the angle measured between the subchondral plate of the distal tibial articular surface and a vertical line to the ground

Radiographic index measurements were carried out using PACS by two experienced fellowship trained adult reconstructive orthopedic surgeons before and after surgery. The radiographic measurements encompassed several aspects: (1) hip-knee-ankle (HKA) angle, defining lower limb alignment as the angle between the mechanical axes of the femur and tibia (Fig. 1). (2) tibiotalar tilt (TTT) angle, which measures the angle between the subchondral plate of the distal tibia articular surface and that of the talar dome; (3) talar inclination, measured between the talar dome and a vertical line to the ground; (4) tibial plafond inclination (TPI), measuring the angle between the subchondral plate of the distal tibial articular surface and a vertical line to the ground (Fig. 2). All radiographs were reviewed by one of 2 fellowship-trained surgeons from the author group. Inter-observer reliability was tested using intra-class correlation coefficient (ICC) with a 2-way random effects model, assuming single measurements and absolute agreement. Sample size for reliability testing was calculated with an intra-class correlation coefficient (ICC) target value of 0.8 and a 95% confidence interval width of 0.2. A minimum number of inter-observer reliability for the 2 raters was 20 by Bonnett’s approximation [8] Thus, a subset of 20 radiographs were read by both the surgeons. As regards interobserver reliability, all radiographic measurements showed excellent ICCs. Preoperative HKA, TPI, TI and TTT ICC were 0.991 (95% CI: 0.985–0.995), 0.972 (95% CI: 0.961–0.989), 0.980 (95% CI: 0.97–0.988) and 0.811 (95% CI: 0.711–0.910) respectively. Postoperative HKA, TPI, TI and TTT ICC were 0.851 (95% CI: 0.98–0.991), 0.973 (95% CI: 0.961–0.989), 0.961 (95% CI: 0.92–0.988) and 0.865 (95% CI: 0.711–0.910) respectively.

### Surgical technique

The femoral and tibial components were virtually positioned according to the balancing principles of tibia first inverse kinematic alignment (iKA) which aims to restore the native tibial joint line while targeting a specific soft tissue balance profile throughout flexion by adjusting the femoral component position from the patient’s native femoral anatomy and allowing some lateral flexion laxity [4, 9]. In every instance, iKA was conducted utilizing imageless robot-assisted navigation employing a tibia-first approach, mirroring the technique outlined by Murgier and Clatworthy and Winnock de Grave et al. [4].

A medial parapatellar approach with minimal medial release during exposure was employed. Optical trackers were affixed to the tibia and femur. Tibial registration involved digitizing the medial and lateral resection depths according to the Murgier and Clatworthy study, utilizing the mid-coronal line of the lateral tibial plateau and a point marked on the tidemark of the medial plateau where cartilage wear measures approximately 2 mm [10]. Femoral anatomy was registered using a 3D morphometric model [11]. An initial kinematic assessment was conducted to determine the range of motion under manual manipulation. Subsequently, the navigation system was used to plan tibial resection, aiming to restore the native joint line in the coronal plane, while considering cartilage wear as described by Murgier and Clatworthy [4, 12], and restricting resection to 5° varus and 3° valgus from the mechanical axis. Tibial resection was performed accordingly using the robotic cutting saw and validated using the navigation system validation pointer with the aim of matching the patient’s native slope within a range of 2°–9°. A mechanical joint tensioner was then introduced into the joint space to collect laxity data through the range of motion prior to secondary balance assessment. The laxity data from the balance assessment were utilized as input for the intraoperative predictive gap-planning software, which virtually positioned the femoral component, providing a postoperative gap prediction throughout flexion. Femoral resections were planned to achieve stability and rectangular mediolateral gaps in extension, while permitting some lateral laxity as the knee transitions into flexion, restricting distal femoral valgus to 3° valgus and 6° varus from the mechanical axis using the predictive gap-planning software. Femoral resections were then carried out using the robotic cutting saw. Final laxity was determined using the implanted tibial insert thickness. Mediolateral balance was characterized as the disparity between lateral and medial laxity. All subsequent measurements were made intraoperatively and recorded into the robotic system user-interface.

### Data analysis

Comparisons between neutral and the severe varus groups were conducted for baseline demographic data and radiographic parameters. Furthermore, comparisons were made between preoperative and postoperative coronal alignment measurements within the two patient groups. Categorical variables were analyzed using the chi-squared test, while descriptive statistics and continuous variables were evaluated using mean, range, and standard deviation. Independent samples t-tests were applied to assess differences in changes from preoperative to postoperative radiographic measurements among the groups. A significance level of *p* < 0.05 was set for all statistical analyses.

## Results

With respect to the radiographic measurements, significant changes in ankle alignment were found in the severe varus ≥ 10° correction group, specifically with regard to TPI (93.7° to 92.2°, *p* = 0.04) and TI (94.9° to 93.4°, *p* = 0.03). In the neutral varus/valgus < 10° correction, there were no significant changes postoperatively. Regardless of the degree of knee deformity correction, iKA TKA in both groups did not lead to significant changes in the TTT angle [Table 2]. When delta change between preoperative and postoperative measurements were compared between groups, only HKA showed a significant difference between groups (*p* = 0.019) [Table 3].

**Table 2:** Average alignment angles of the knee and ankle measured before and after surgery using low-radiation, full-length standing anteroposterior imaging in patients who underwent total knee arthroplasty. Data is presented as mean (standard deviation)

	Preoperative (± SD)	Postoperative (± SD)	Δ	*p*-value
Neutral varus < 10° (*N* = 87)				
Hip-knee-ankle angle	3.6 (± 4.8)	2.8 (± 3.3)	-0.8 (± 4.3)	0.07
Tibial plafond inclination angle	89.2 (± 6.6)	90 (± 4.7)	-1.0 (± 4.8)	0.06
Talar inclination angle	90.3 (± 7.2)	91 (± 5.0)	-0.7 (± 5.2)	0.24
Tibiotalar tilt angle	1.7 (± 1.6)	1.7 (± 1.6)	0.0 (± 1.2)	0.93
Severe varus ≥ 10° (*N* = 36)				
Hip-knee-ankle angle	14.5 (± 2.7)	6.4 (± 2.7)	-8.1 (± 2.7)	**< 0.001**
Tibial plafond inclination angle	93.7 (± 6.1)	92.2 (± 5.0)	1.5 (± 4.4)	**0.04**
Talar inclination angle	94.9 (± 6.5)	93.4 (± 5.0)	1.5 (± 4.0)	**0.03**
Tibiotalar tilt angle	1.68 (± 1.8)	1.9 (± 1.5)	-0.2 (± 1.4)	0.41

**Table 3:** Comparison of Δ change in measured angles between before and after total knee arthroplasty across groups

	Neutral varus < 10° (*N* = 87)	Severe varus ≥ 10° (*N* = 36)	*p*-value
Δ change from pre to postoperative			
Hip-knee-ankle angle	-0.8 (± 4.3)	-8.1 (± 2.7)	**0.019**
Tibial plafond inclination angle	-1.0 (± 4.8)	1.5 (± 4.4)	0.90
Talar inclination angle	-0.7 (± 5.2)	1.5 (± 4.0)	0.15
Tibiotalar tilt angle	0.0 (± 1.2)	-0.2 (± 1.4)	0.3

## Discussion

The findings of this study demonstrate that correction of ≥ 10° varus knee deformity (HKA) can significantly influence ankle joint alignment postoperatively, particularly leading to alterations in the TPI and talar inclination. This phenomenon was not observed in the neutral varus group.

Full-length lower extremity radiographs are the gold standard for assessing overall limb alignment, providing a thorough evaluation of the hip-knee alignment [13]. Despite such imaging being the benchmark for evaluating TKA candidates preoperatively, pathological and adaptational alterations in the ankles and hips are generally overlooked. Varus or valgus alignment deformity of knee can cause malalignment of the ankle and affect the ankle tilt. Ariywatkul et al. demonstrated that alteration of 14.5° or more in femorotibial anatomical knee axis escalates the risk for reduced parallelism of the ankle joint line [6]. Concurrently, some studies described a 24–35% rate of simultaneous knee and ankle pathologies, with one revealing a 22% developing progressive ankle arthritis within at least 3 years after TKA [3, 14, 15]. Xie et al. revealed that relative valgus tilt of the talus and distal tibia plafond was aggravated when varus knee deformities advanced [16]. Norton et al. further demonstrated that varus knee deformity resulted in subsequent valgus hindfoot alignment, and vice versa, with valgus knees developing varus hindfoot alignments [17]. This could be attributed to knee deformities provoking abrupt change in the biomechanics and amplifying tilt in an already degenerated ankle [10, 12, 18]. While some studies reported an aggravation of normal or valgus ankle alignment, others revealed improvement following correction of knee malalignment following TKA [12, 18]. Therefore, providing an inconclusive association between knee malalignment and concomitant ankle morphology change [3, 19, 20].

A study by Chang et al. reported an increase of 1.4° in TTT angle, called ankle varus incongruency, following correction of > 10 degrees genu varum deformity after TKA [21]. These results were consistent with the review by Feng et al., where knee varus correction was correlated with improved postoperative ankle coronal malalignment [22]. On the other hand, this study did not demonstrate significant change in TTT in both neutral and severe varus groups. A recent study by Jin et al. demonstrated a change in TTT in correction of varus deformities ≥ 10°, representing varus incongruence exacerbation [23]. A contemporary systemic review by Oevelen et al. showed that the biomechanical and clinical outcome improvements following knee osteotomies at the level of the ankle/ hindfoot could be adversely impacted by a rigid subtalar joint, small preoperative lateral distal tibial angle (LDTA) and HKA or a large post-operative HKA corrections [19].

Shichman et al. [24] concluded that preoperative full-length standing lower extremity radiographs, which included ankle alignment, aided in anticipating adaptational changes and expected development or aggravation of ankle pain postoperatively. However, they did not find a relationship between TPI and TI in varus deformities regardless of the severity of knee deformity. Alternatively, our results were consistent with regards to neutral varus correction where no significant change was found in ankle alignment. On the contrary, a significant decrease in TPI (93.7 to 92.2, *p* = 0.04) and TI (94.9 to 93.4, *p* = 0.03) angles was found following severe varus correction, which was consistent with Chong Bum et al. where a significant decrease was demonstrated in TPI (100.4 to 93.2, *p* < 0.001) and TI (103.5 to 97.8, *p* < 0.001) following mechanical TKR of ≥ 10° varus correction [21].

Though ankle symptoms were not collected due to the design of this study, we believe that iKA was an important contributing factor to preserving or minimizing substantial coronal alignment changes of the ankle joint. A larger cohort however may still be needed to further solidify these findings as TPI change in the neutral varus/valgus < 10° correction group was near significance (*p* = 0.06).

This study is subject to various limitations that need to be addressed. To begin with, this is a retrospective study where several biases including observer and selection biases could have arisen and influenced study outcomes. Secondly, the study’s primary emphasis lies in assessing coronal alignment exclusively, which raises a fundamental limitation from attempting to measure a 3-dimensional entity within the boundaries of a 2-dimensional framework. Furthermore, this study is limited by plain X-ray imaging, where rotational elements and image quality could have drastically influenced the measurement of deformities and subsequent categorization. Furthermore, analysis of ankle radiographic changes was restricted solely to the tibiotalar joint due to inadequate visibility in standing radiographs to assess the sub-talar joint. This could serve as a pivot for the ankle joint compensation mechanism and should be evaluated in future studies. Additionally, our cohort included only varus knee deformities, hence, this study findings should be interpreted to varus knees alone and future investigation is warranted to assess ankle coronal alignment changes in valgus knee deformities. Finally, our data solely focused on the radiographic evaluation with omission of clinical ankle symptoms. Hence, the relationship between the radiographic alterations and their impact on clinical ankle-related symptoms still remains unclear.

## Conclusion

Inverse kinematic alignment TKA of varus knee deformity preserves or minimizes substantial coronal alignment changes of the ankle joint in patients with neutral (< 10° varus) preoperative knee deformities. These findings may add to the benefits of patient specific alignment TKA techniques. The findings of this study also remind surgeons of the primary objectives of TKA in restoring mobility, in addition to enhancing patient satisfaction and improving quality of life. Thus, our study highlights the importance of preoperative evaluation using standing long leg images in order to calculate the desired alignment best suited for meeting the expectations of the patient postoperatively.
