# Correction: Effect of inverse kinematic alignment total knee arthroplasty on coronal alignment of the ankle joint in patients with varus knee deformity

**DOI:** 10.1007/s00402-024-05748-x

**Published:** 2025-01-17

**Authors:** Ittai Shichman, Amer Hallak, Itay Ashkenazi, Yaniv Warschawski, Aviram Gold, Nimrod Snir

**Affiliations:** 1 Division of Orthopedic Surgery, Tel-Aviv Sourasky Medical Center, Tel-Aviv, Israel; 2 Chief of Adult Reconstruction Unit, Division of Orthopedic Surgery, Tel Aviv Sourasky Medical Center, NYU Langone Orthopedic Center, 6 Weizman St. 6th Floor, Tel-Aviv, Israel

**Correction to: Archives of Orthopaedic and Trauma Surgery (2024) 144:4455-4461**

10.1007/s00402-024-05549-2

In the original version of this article, the author’s name Yaniv Warschawski was incorrectly written as Yaniv Warschwaski.

